# A systems immunology perspective on gout pathogenesis and its precision-targeted treatment strategies

**DOI:** 10.3389/fimmu.2025.1615914

**Published:** 2025-06-19

**Authors:** Zilong Chen, Qian Guo, Yanzhao Zhang, Lulu Chen, Puyu Li, Wenfei Cheng, Chuanxin Liu, Hongwei Jiang

**Affiliations:** 1 Endocrinology and Metabolism Center, The First Affiliated Hospital, and College of Clinical Medicine of Henan University of Science and Technology, Luoyang, China; 2 Department of Rhinology, The First Affiliated Hospital of Zhengzhou University, Zhengzhou, China

**Keywords:** gouty arthritis, autophagy, autoimmune, immunotherapy, cytokine, monosodium urate crystals

## Abstract

Gouty arthritis (GA) is a sterile inflammatory disease driven by monosodium urate (MSU) crystal deposition, which activates innate and adaptive immune responses. Key mechanisms involve NLRP3 inflammasome activation, cytokine release (IL-1β, TNF-α, IL-6), and dysregulated autophagy, positioning GA at the intersection of metabolic and autoimmune disorders. While conventional therapies (colchicine, NSAIDs) remain first-line, their limitations in refractory cases have spurred the development of biologic agents targeting pro-inflammatory pathways. Clinical studies demonstrate that TNF-α inhibitors (etanercept, infliximab), IL-6 blockade (tocilizumab), and autophagy modulators effectively reduce flares and inflammation in treatment-resistant GA. Emerging strategies, including combination therapies and biomarker-guided approaches, highlight the shift toward precision medicine in GA management. This review summarizes current insights into GA’s immunopathogenesis and evaluates the therapeutic potential of immunomodulatory biologics.

## 1 Introduction

Gouty arthritis (GA) arises when serum uric acid exceeds the saturation threshold, leading to monosodium urate (MSU) crystal formation and deposition in joint tissues. These crystals trigger an innate immune response by recruiting neutrophils and inducing inflammatory cytokine production (1, 2). Crosstalk among metabolic, immune, and inflammatory pathways drives GA onset and progression, prompting its redefinition as an autoimmune inflammatory disorder (3, 4). Autophagy also plays a dual role by regulating inflammation through NLRP3 inflammasome secretion and innate immune modulation (5–7).

Clinically, colchicine, NSAIDs, and glucocorticoids remain first-line therapies for acute GA but are often limited by adverse effects and high relapse rates (8, 9). With deeper insights into GA pathogenesis, especially the roles of cytokines and immune pathways, various biologic agents targeting inflammation and immune modulation have been introduced into clinical practice. These therapies aim to inhibit cytokine signaling and downstream inflammatory cascades during acute flares, suppress uric acid-induced immune responses, and relieve symptoms, offering alternative options for patients unresponsive or intolerant to conventional treatments. This review summarizes current knowledge on GA’s immunological mechanisms and recent advances in immunotherapy.

## 2 Immunological pathogenesis of gouty arthritis

### 2.1 Role of innate immunity in gouty arthritis

#### 2.1.1 Activation and function of innate immune cells

Innate immune cells, including monocytes/macrophages, mast cells, and neutrophils, play pivotal roles in initiating acute gouty inflammation. Monocytes and macrophages, integral to innate immunity, contribute significantly to arthritis pathophysiology (10). Upon activation, they release pro-inflammatory cytokines such as IL-6, IL-8, TNF-α, and IL-1β. Neutrophils residing in joints, upon encountering MSU crystals, undergo activation, phagocytose crystals, and release inflammatory mediators including IL-1β, TNF-α, and IL-6, thereby intensifying acute inflammatory gout flares.

#### 2.1.2 Dynamics of inflammatory cell recruitment and activation

MSU crystals deposit in joint structures during hyperuricemia, remaining inert until external factors (cold, trauma, alcohol) induce their release into the joint cavity, triggering inflammation (11). During acute gout attacks, mast cells and monocytes/macrophages activate prior to neutrophil infiltration (12). Mast cells rapidly release histamine, serotonin (5-HT), proteases, eicosanoids, IL-1, and IL-8, leading to platelet aggregation, edema, enhanced neutrophil oxidative burst, increased leukocyte-endothelial adhesion, and amplified cytokine release (13–15). Activated monocytes/macrophages produce IL-1, TNF-α, MCP-1, thus stimulating phospholipase A2 and nitric oxide synthase activity in neutrophils and endothelial cells, exacerbating inflammation while inhibiting apoptosis (16). Neutrophils express adhesion molecules such as Mac-1 and secrete inflammatory cytokines, including IL-1β, IL-8, TNF-α, MIP-1 and enzymes (myeloperoxidase, proteases), perpetuating tissue injury (16–18). In a rat air pouch model using 10 mg MSU crystals, Schiltz et al. observed that inflammation reached its peak at 24 hours and completely resolved by day 3 (19). The study revealed distinct temporal patterns of immune cell infiltration: mast cells showed early activation at 1–2 hours, monocytes and macrophages exhibited a sharp 160% increase by 2 hours that persisted for 48 hours, while neutrophils displayed biphasic peaks at 4 and 24 hours.

#### 2.1.3 Monocyte-to-macrophage differentiation and inflammation resolution

Mature macrophages play a pivotal role in modulating the progression from asymptomatic hyperuricemia to acute gouty arthritis (20). During flare episodes, circulating monocytes infiltrate inflamed joints and undergo differentiation into macrophages, which actively phagocytose monosodium urate (MSU) crystals, downregulate the expression of key pro-inflammatory mediators such as TNF-α and ICAM-1, and secrete anti-inflammatory factors including TGF-β, prostaglandin E_2_, and platelet-activating factor to promote the resolution of inflammation (21). In contrast, immature monocytes exhibit a pronounced pro-inflammatory profile, producing elevated levels of TNF-α, IL-1, and IL-6, and promoting neutrophil recruitment through upregulation of endothelial E-selectin expression (22). Research by So et al. demonstrated that partially differentiated macrophages are characterized by high TNF-α secretion and robust ICAM-1 induction, both of which can be attenuated through TNF-α blockade. Fully differentiated macrophages, on the other hand, predominantly function in MSU crystal clearance with minimal cytokine output (23). These findings underscore the dynamic and stage-specific roles of monocytes and macrophages in gout pathogenesis, highlighting the coordinated interplay among mast cells, monocyte/macrophage subsets, and neutrophils in orchestrating both the initiation and resolution of gouty inflammation.

### 2.2 Role of immunoglobulins in gouty arthritis

The interaction between monosodium urate (MSU) crystals and immunoglobulins represents a critical regulatory mechanism in gouty inflammation. During the initial inflammatory phase, MSU crystals bind to the Fab region of IgG, forming immune complexes that activate innate immune responses. This IgG-mediated opsonization enhances crystal phagocytosis by neutrophils through Fcγ receptor engagement, leading to lysosomal enzyme release and subsequent inflammatory amplification (24). However, this pro-inflammatory effect exhibits concentration-dependent regulation, as excessive IgG adsorption on MSU crystals ultimately disrupts further immune activation through lysosomal membrane destabilization and hydrolytic enzyme release (25). The resolution phase involves a dynamic shift in MSU crystal surface protein composition. While IgG dominates during acute inflammation, high-affinity binding of apolipoprotein B (apoB) becomes predominant during resolution. ApoB competitively inhibits IgG-MSU interactions, thereby suppressing leukocyte phagocytic activity, reactive oxygen species production, and pro-inflammatory cytokine secretion. Similarly, other plasma proteins including fibrinogen and fibronectin contribute to inflammation resolution by attenuating chemokine release and leukocyte oxidative bursts. Clinical observations demonstrate a strong correlation between synovial fluid IgG-MSU deposition and elevated leukocyte counts, whereas apoB enrichment coincides with inflammatory resolution (26). These findings are further supported by experimental models showing that temporal changes in MSU crystal protein coating directly regulate the transition from pro-inflammatory to anti-inflammatory states (27).

### 2.3 Cytokine-mediated regulation of gouty inflammation

TGF-β mediates immunosuppressive effects by inhibiting lymphocyte proliferation and antagonizing pro-inflammatory cytokines, crucial for inflammation resolution (25). Shi et al. reported that recombinant human TGF-β1 significantly reduced MSU-induced leukocyte infiltration and monocyte accumulation. TGF-β1 antagonists reversed these effects, validating its anti-inflammatory function. Additionally, MSU crystals upregulate monocyte/macrophage-derived TGF-β, suppressing neutrophil recruitment via E-selectin inhibition (25). Tumor necrosis factor α (TNF-α) is pivotal in gout pathogenesis. Busso et al. demonstrated that TNF-α blockade reduces E-selectin expression and neutrophil recruitment in MSU-induced arthritis (26). Matsukawa et al. found elevated TNF-α mRNA expression in MSU-stimulated synovial monocytes, enhancing neutrophil-mediated IL-1 release (27). Stefanie et al. revealed TNF-α-driven neutrophil activation, caspase-1 secretion, and subsequent IL-1β cleavage and activation, further amplifying inflammatory responses. Elevated TNF-α levels in GA patients’ serum and synovial fluid compared to healthy controls emphasize its clinical relevance and therapeutic potential (28).

### 2.4 NOD-like receptors and inflammasome activation in GA pathogenesis

Toll-like receptors (TLRs), located on cellular membranes, are critical pattern recognition receptors within the innate immune system. These receptors serve as vital sensors, identifying pathogenic and danger-associated molecular patterns (DAMPs). Notably, MSU crystals specifically interact with TLR2, TLR4, and the surface adapter molecule CD14. Upon binding to these receptors, MSU crystals trigger downstream signaling via the adaptor protein MyD88, leading to IL-1βrelease and neutrophil infiltration, ultimately contributing to the inflammatory cascade characteristic of gout arthritis (GA) (29, 30). NOD-like receptors (NLRs), cytoplasmic counterparts of TLRs, function as intracellular sensors recognizing endogenous danger signals as well as exogenous pathogens. Recent advances have highlighted their significant roles in the immune responses associated with GA. Specifically, the NLRP3 inflammasome, a prominent NLR family member, is activated by MSU crystals, initiating a robust inflammatory response marked by elevated production of IL-1β (31). Experimental studies using murine models indicate that mice deficient in NLRP3 fail to produce IL-1β upon exposure to MSU crystals, underscoring the essential role of this inflammasome complex in GA pathogenesis (32).

### 2.5 Adaptive immunity in GA pathogenesis

The adaptive immune system, characterized by its antigen-specific recognition, clonal diversity, and immunological memory, primarily operates through the coordinated actions of T and B lymphocytes. MSU crystals can directly participate in immune cells and contribute to gouty arthritis (GA) (33). Notably, these crystalline structures demonstrate the capacity to activate autoreactive T cell clones in an antigen-presenting cell (APC)-independent manner, suggesting a novel mechanism by which adaptive immunity may perpetuate inflammatory cascades in GA pathogenesis (34). B lymphocytes and immunoglobulins have also been implicated in GA inflammation. Studies involving B cell-deficient mice reveal that the absence of B cells substantially diminishes the inflammatory response to urate crystals. Conversely, the administration of immunoglobulin M (IgM) restores the inflammatory potential of urate, significantly amplifying the basal inflammatory state (35).

## 3 Autophagy-mediated immune regulation in gouty arthritis

### 3.1 Autophagy and innate immune responses

**Figure 1:**
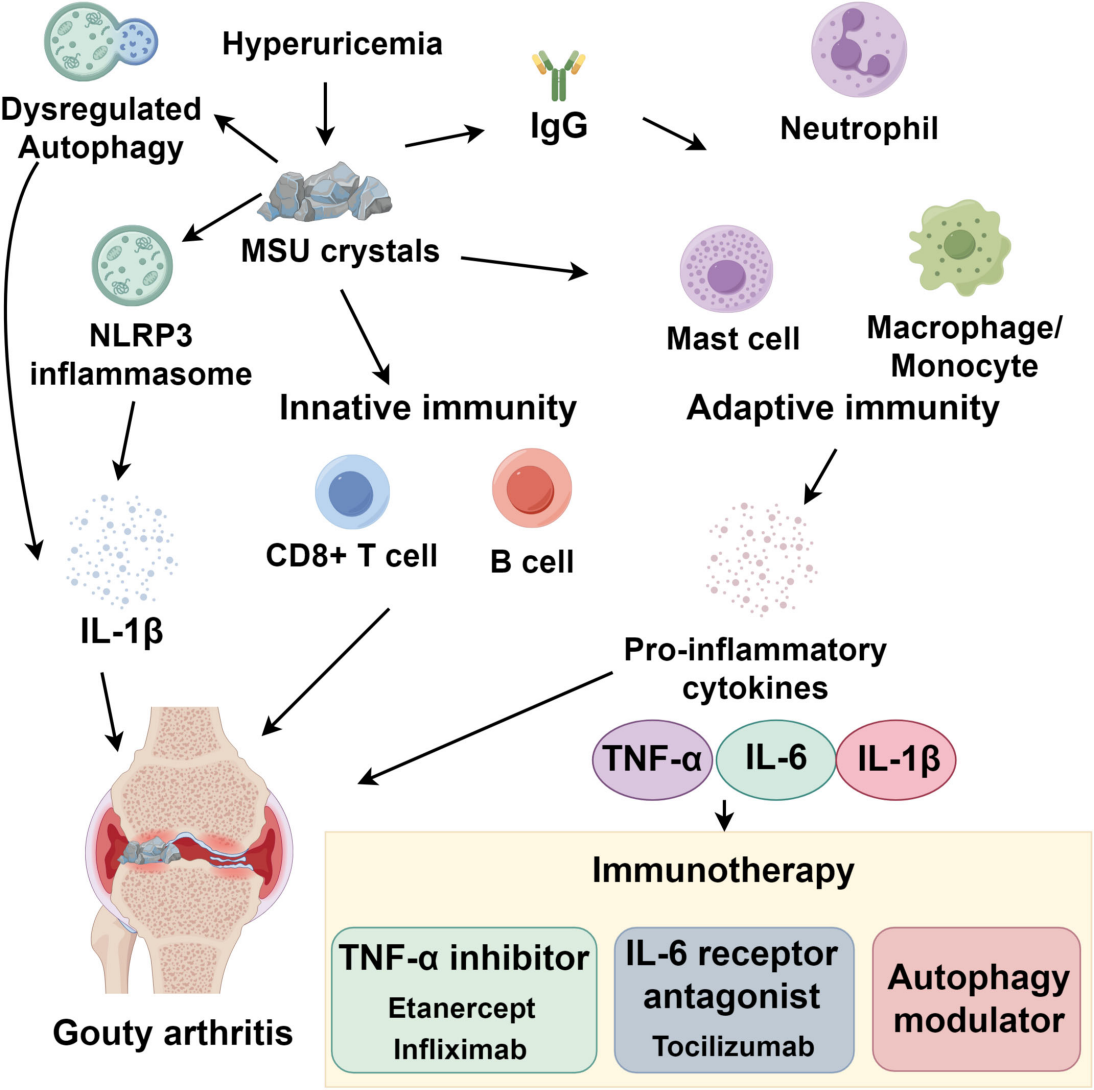
Integrated immune mechanisms driving gouty arthritis and therapeutic intervention strategies.

Innate immune receptors (TLRs/NLRs) stimulate autophagic activity. Stimulation of TLR4 or TLR7 promotes autophagosome biogenesis, which facilitates mycobacterial elimination (36). NLR family proteins orchestrate NLRP3 inflammasome formation, caspase-1 stimulation, and pyroptotic cell death—all negatively regulated by autophagic processes (37). The protein DRAM1 functionally connects TLR-MyD88-NF-κB cascades to selective autophagy induction (38). Within adaptive immunity, autophagy governs antigen processing (MHC I/II pathways), lymphocyte maturation, and cytokine release. Exogenous antigens undergo cross-presentation on MHC I molecules through autophagic mechanisms, whereas MHC II molecules present cytosolic antigens via peptides derived from autophagosomal compartments (39). Pathogen-derived antigens degraded within autophagosomes are transported to MHC II-loading vesicles, activating CD4^+^ and CD8^+^ T cells (39, 40). Phagocytic uptake coupled with LC3/ATG8 conjugation augments extracellular antigen presentation via MHC II (41). Additionally, γδT-cell secretion of IL-17, TNF-γ, and IL-22 is modulated by autophagy, highlighting its immunomodulatory functions (42) (**Figure 1**).

### 3.2 Autophagic mechanisms in GA pathogenesis

Elevated autophagic activity and pathway induction are observed in acute GA patients (43). *In vitro*, monosodium urate (MSU) crystals simultaneously stimulate NLRP3 inflammasome activation and autophagy upregulation (44). Neutrophils exposed to MSU exhibit increased LC3-II levels, an effect abolished by the autophagy inhibitor 3-MA (45). The anti-inflammatory cytokine TGF-β1 regulates autophagic flux through diverse pathways (46). Monocyte-to-macrophage differentiation during GA resolution produces TGF-β1, a pivotal mediator of inflammatory suppression (47). Autophagy exhibits dual roles in GA inflammation: While Shi et al. (48) demonstrated its inhibitory effect on MSU-induced IL-1β production, Choe et al. (49) reported that MSU-induced autophagosomes fail to degrade ubiquitinated substrates, leading to p62 accumulation and exacerbated IL-1β secretion. Therapeutic targeting of autophagy—via apoptosis modulation, immune reprogramming, or anti-inflammatory effects—holds clinical promise. Pharmacological agents, such as rapamycin, resveratrol, chloroquine) are employed in oncology, transplantation, and infectious diseases. In GA models, resveratrol counteracts MSU-mediated SIRT1 suppression in a dose-dependent manner, restoring autophagic flux and attenuating inflammation (50). SIRT1 induction represents a critical control point, positioning autophagy modulators (activators or inhibitors) as viable therapeutic options (**Table 1**).

**Table 1:** Immunological mechanisms in gouty arthritis (GA) pathogenesis.

Mechanism	Key Players	Pathological Effects	Therapeutic Targeting
Activation of Innate Immune Cells	Monocytes, macrophages, neutrophils, mast cells	Release of pro-inflammatory cytokines (IL-1β, TNF-α, IL-6), phagocytosis of MSU crystals, inflammatory cascade	Inhibition of cytokine signaling (e.g., TNF-α, IL-1β blockers), autophagy modulation
NLRP3 Inflammasome Activation	NLRP3, caspase-1, IL-1β	Activation of inflammasome by MSU crystals leading to IL-1β release and neutrophil infiltration	NLRP3 inflammasome inhibitors, autophagy activators or inhibitors
Autophagy and Inflammation Resolution	Autophagy receptors (LC3, ATG), TGF-β1	Dual role: inhibition of IL-1β production, regulation of inflammation resolution	Targeting autophagy flux (rapamycin, resveratrol, chloroquine) to modulate immune response
Monocyte-to-Macrophage Differentiation	Monocytes, macrophages, TNF-α, ICAM-1	Differentiation into pro-inflammatory macrophages, cytokine release	Anti-TNF-α antibodies, macrophage polarization therapy
Immunoglobulins and MSU Crystals	IgG, IgM, apoB, fibrinogen, fibronectin	IgG opsonization promotes MSU crystal phagocytosis and inflammation	IgG-mediated immune modulation, targeting MSU-IgG complex formation
Cytokine-Mediated Inflammation	TNF-α, IL-1β, IL-6, TGF-β, MCP-1	Pro-inflammatory cascade via TNF-α and IL-1β, anti-inflammatory via TGF-β	TNF-α inhibitors, IL-6 receptor antagonists, TGF-β1 mimetics
Role of Adaptive Immunity	T-lymphocytes (CD8^+^, CD4^+^), B-lymphocytes, MSU crystals	Autoreactive T cells and B cells exacerbate inflammation	B-cell depletion (e.g., rituximab), CD8^+^ T cell modulation

## 4 Therapeutic immunomodulation in gouty arthritis

### 4.1 Targeted TNF-α blockade

Etanercept, a recombinant fusion protein comprising the extracellular ligand-binding region of human TNF receptor 2 coupled to IgG1-Fc, exhibits superior TNF-α binding capacity compared to endogenous receptors. This biologic agent sequesters soluble TNF-α, preventing receptor engagement and subsequent proinflammatory signaling, while the Fc moiety enhances pharmacokinetic stability. Clinical evidence from Tausche et al. (51) describes a refractory GA patient showing no response to conventional therapies, including colchicine, NSAIDs, steroids, and opioids. Twice-weekly subcutaneous etanercept markedly decreased flare frequency, tender joint count and inflammatory markers. A separate case by Zhang et al. (52) involved a 48-year-old male with treatment-resistant tophaceous gout, hypertension, and infected ulcers. Following etanercept initiation, rapid symptomatic relief preceded full ambulatory recovery by 14 d, with normalized CRP/ESR and serum urate. Long-term febuxostat/colchicine therapy achieved tophi clearance within 12 months without disease recurrence. Adalimumab, as a human IgG1 monoclonal antibody targeting TNF-α, demonstrates an extended plasma half-life and lacks immunogenic animal-derived epitopes. Sumiyoshi et al. (53) detailed its application in a 39-year-old male with HLA-B27–associated reactive arthritis secondary to C. trachomatis infection, superimposed on chronic gout. Despite maximal anti-inflammatory therapy, disease progression occurred. Combination therapy with sulfasalazine and biweekly adalimumab induced prompt clinical remission. Serial ultrasonography confirmed complete resolution of synovial and tendinous inflammation by 12 months (53).

Infliximab exhibits high specificity for transmembrane TNF-α. Upon binding, it triggers cell lysis through complement-dependent cytotoxicity (CDC) and antibody-dependent cellular phagocytosis (ADCP). Fiehn and Zeier (54) reported its therapeutic application in a 44-year-old male with refractory gouty arthropathy and secondary renal amyloidosis. Symptomatic recurrence at six weeks necessitated repeat infusion, which again induced prompt clinical resolution. Maintenance therapy demonstrated excellent tolerability with durable therapeutic effects.

### 4.2 IL-6 pathway inhibition in refractory gout management

The humanized monoclonal antibody tocilizumab (TCZ) targets both soluble and membrane-bound IL-6 receptors, effectively disrupting downstream gp130 signaling cascades. Clinical evidence demonstrates its therapeutic potential in treatment-resistant gout cases. Pinto and colleagues (55) reported on a 44-year-old male patient with advanced tophaceous gout who exhibited poor response to conventional therapies, including colchicine, allopurinol, and diclofenac. Although monthly tocilizumab (TCZ) infusions at a dose of 8 mg/kg were effective in preventing acute gout flares, they did not lead to a marked reduction in tophus burden (55). In a separate case described by Mokuda et al. (56), a 61-year-old woman with concomitant gout and renal dysfunction achieved complete clinical remission following TCZ treatment administered every four weeks at the same dosage. The intervention resulted in normalization of inflammatory biomarkers, including C-reactive protein and ferritin, and sustained disease control over a 24-week period, despite prior failure of colchicine combined with prednisone. Calvo-Aranda and Sanchez-Aranda (57) reported favorable therapeutic outcomes following tocilizumab (TCZ) treatment in a 56-year-old male patient with refractory gout and coexisting metabolic conditions, including hypertriglyceridemia and hyperferritinemia, after inadequate response to prior anakinra therapy. The treatment achieved rapid symptom control with maintained efficacy throughout 12 months of follow-up, exhibiting an excellent safety profile. Pers et al. (58) evaluated tocilizumab (TCZ) in elderly rheumatoid arthritis patients, finding that while it was well tolerated, its efficacy was lower compared to younger patients. Despite this, TCZ led to sustained clinical improvement over 6 months with good tolerability. In a prospective study, Latourte et al. (59) assessed the efficacy of tocilizumab (TCZ) in 11 patients with calcium pyrophosphate deposition (CPPD) disease who had shown resistance to multiple therapeutic agents, including colchicine, nonsteroidal anti-inflammatory drugs, corticosteroids, and anakinra. Both intravenous and subcutaneous regimens showed universal clinical improvement by 3 months, with durable responses maintained through 10 months of follow-up. The treatment was generally well-tolerated, with only three reported adverse events.

## 5 Emerging directions: microbiome and epigenetic regulation in GA

### 5.1 Microbiome contributions to gouty inflammation

Recent studies suggest that dysbiosis of the gut microbiota may modulate systemic inflammation and urate metabolism, offering a novel perspective on GA pathogenesis (60). Gut microbes influence purine metabolism by regulating the synthesis of purine-metabolizing enzymes (61). Patients with gout often exhibit decreased levels of *Faecalibacterium prausnitzii* and *Bifidobacterium*, both known for their anti-inflammatory properties and short-chain fatty acid (SCFA) production (62, 63). SCFAs such as butyrate inhibit histone deacetylases (HDACs), promoting regulatory T cell (Treg) differentiation and attenuating NLRP3 inflammasome activation (64, 65). Moreover, fecal microbiota transplantation (FMT) and probiotic supplementation have demonstrated preliminary success in reducing systemic inflammation and uric acid levels in experimental models (66). These findings underscore the therapeutic potential of microbiota modulation in GA management.

### 5.2 Epigenetic modulation of inflammatory pathways

Epigenetic alterations, including DNA methylation, histone modifications, and non-coding RNA expression, critically regulate gene transcription in immune and metabolic pathways implicated in GA (67). Hypermethylation of promoter regions in anti-inflammatory genes such as IL-10, and hypomethylation in pro-inflammatory loci, such as TNF, have been identified in patients with chronic gout (68). Histone acetylation is particularly relevant: reduced histone H3 acetylation at the FOXP3 locus compromises Treg cell function and amplifies joint inflammation (69, 70). Inhibitors targeting epigenetic enzymes, such as DNA methylation, have shown potential immunosuppressive and uric-lowering effects (71, 72). Additionally, microRNAs (miRNAs), especially miR-155 and miR-146a, modulate TLR/NF-κB signaling and IL-1β secretion in GA, further supporting the role of non-coding RNA-based regulation (73–76).

## 6 Conclusion

Gouty arthritis (GA) represents a complex interplay between metabolic dysfunction and immune dysregulation, driven by monosodium urate (MSU) crystal deposition and subsequent inflammatory cascades. The immunopathogenesis of GA involves innate and adaptive immune responses, including NLRP3 inflammasome activation, cytokine release, and dysregulated autophagy. These mechanisms highlight GA as a paradigm of sterile inflammation with autoimmune features. While traditional therapies like colchicine and NSAIDs remain first-line, their limitations in refractory cases have spurred the development of targeted biologics. Agents such as TNF-α inhibitors (etanercept, infliximab), IL-6 blockers (tocilizumab), and autophagy modulators have demonstrated efficacy in reducing inflammation and flare frequency, offering promising alternatives for treatment-resistant patients.

Emerging research on microbiome dysbiosis and epigenetic regulation further expands the therapeutic landscape. Gut microbiota alterations influence urate metabolism and systemic inflammation, while epigenetic modifications modulate immune responses in GA. These insights pave the way for precision medicine approaches, including biomarker-guided therapies and combination strategies. Future studies should focus on optimizing biologic use, exploring novel immunomodulatory targets, and integrating microbiome/epigenetic interventions to improve long-term outcomes. By elucidating GA’s intricate immunology, this review underscores the shift toward personalized, mechanism-based management for this debilitating conditions.
