# Correction: A systems immunology perspective on gout pathogenesis and its precision-targeted treatment strategies

**DOI:** 10.3389/fimmu.2025.1713414

**Published:** 2025-11-06

**Authors:** Zilong Chen, Qian Guo, Yanzhao Zhang, Lulu Chen, Puyu Li, Wenfei Cheng, Chuanxin Liu, Hongwei Jiang

**Affiliations:** 1 Endocrinology and Metabolism Center, The First Affiliated Hospital, and College of Clinical Medicine of Henan University of Science and Technology, Luoyang, China; 2 Department of Rhinology, The First Affiliated Hospital of Zhengzhou University, Zhengzhou, China

**Keywords:** gouty arthritis, autophagy, autoimmune, immunotherapy, cytokine, monosodium urate crystals

Reference “30” was not cited in the article. The citation has now been inserted in the scetion **2 Immunological pathogenesis of gouty arthritis**, *2.4 NOD-like receptors and inflammasome activation in GA pathogenesis* , 4 and should read:

“Upon binding to these receptors, MSU crystals trigger downstream signaling via the adaptor protein MyD88, leading to IL-1βrelease and neutrophil infiltration, ultimately contributing to the inflammatory cascade characteristic of gout arthritis (GA) (29, 30)”

Reference “16” was erroneously written as “Posada Arias S, Rey-Suárez P, Pereáñez JA, Acosta C, Rojas M, Delazari Dos Santos L, et al. Isolation and functional characterization of an acidic myotoxic phospholipase A2 from Colombian bothrops asper venom. Toxins (Basel). (2017) 9:342. doi: 10.3390/toxins9110342”. It should be “Shirai T, Hilhorst M, Harrison DG, Goronzy JJ, Weyand CM. Macrophages in vascular inflammation–From atherosclerosis to vasculitis. Autoimmunity. (2015) 48:139-151. doi:10.3109/08916934.2015.1027815”.

Reference for “22” was erroneously written as “Yagnik DR, Florey O, Philippidis P, Emons V, Mason JC, Haskard DO, et al. Safe disposal of inflammatory monosodium urate monohydrate crystals by differentiated macrophages. Arthritis Rheumatol. (2002) 46:3026–33. doi: 10.1002/art.v46:11”. It should be “Yagnik DR, Florey O, Philippidis P, Emons V, Mason JC, Haskard DO, et al. Safe disposal of inflammatory monosodium urate monohydrate crystals by differentiated macrophages. Arthritis Rheumatol. (2002) 46:3026–33. doi: 10.1002/art.10614”.

Reference for “23” was erroneously written as “Renaudin F, Orliaguet L, Castelli F, Fenaille F, Prignon A, Alzaid F, et al. Gout and pseudo-gout-related crystals promote GLUT1-mediated glycolysis that governs NLRP3 and interleukin-1β activation on macrophages. Ann Rheum Dis. (2020) 79:1506–14. doi: 10.1136/annrheumdis-2020-217342”. It should be “So AK, Martinon F. Inflammation in gout: mechanisms and therapeutic targets. Nat Rev Rheumatol. (2017) 13:639-647. doi:10.1038/nrrheum.2017.155”.

Reference “25” was erroneously written as “Russell IJ, Papaioannou C, McDuffie FC, MacIntyre S, and Kushner I Effect of IgG and C-reactive protein on complement depletion by monosodium urate crystals. J Rheumatol. (1983) 10:425–33”. It should be “Shi M, Luo J, Ding L, Duan L. Spontaneous resolution of acute gout: mechanisms and therapeutic targets. RMD Open. (2023) 9:e003586. doi:10.1136/rmdopen-2023-003586”.

Reference “26” was erroneously written as “Jung J, Bong JH, Lee SJ, Kim MJ, Sung JS, Lee M, et al. Screening of fv antibodies with specific binding activities to monosodium urate and calcium pyrophosphate dihydrate crystals for the diagnosis of gout and pseudogout. ACS Appl Bio Mater. (2021) 4:3388–97. doi: 10.1021/acsabm.0c01680”. It should be “Busso N, So A. Mechanisms of inflammation in gout. Arthritis Res Ther. (2010) 12:206. doi:10.1186/ar2952”.

Reference “27” was erroneously written as “Type II collagen facilitates gouty arthritis by regulating MSU crystallisation and inflammatory cell recruitment. Ann Rheum Dis. (2023) 82:416–27. doi: 10.1136/ard-2022-222764”. It should be “Scanu A, Oliviero F, Ramonda R, Frallonardo P, Dayer JM, Punzi L. Cytokine levels in human synovial fluid during the different stages of acute gout: role of transforming growth factor β1 in the resolution phase. Ann Rheum Dis. (2012) 71:621-624. doi:10.1136/annrheumdis-2011-200711”.

Reference “32” was erroneously written as “Sharma K, Dresser K, and Shi Y. A role of lgM antibodies in monosodium urate crystal formation and associated adjuvanticity. Immunol. (2009) 182:1912–8. doi: 10.4049/iimmunol.0803777”. It should be “Martinon F, Pétrilli V, Mayor A, Tardivel A, Tschopp J. Gout-associated uric acid crystals activate the NALP3 inflammasome. Nature. (2006) 440:237-241. doi:10.1038/nature04516”.

Reference “33” was erroneously written as “Elmaoued RA, Davis AS, Kyei G, and Deretic V. Toll-like receptors control autophagy. EMBO J. (2008) 27:1110–21. doi: 10.1038/emboi.2008.31”. It should be “Zamudio-Cuevas Y, Fernández-Torres J, Martínez-Nava GA, et al. Phagocytosis of monosodium urate crystals by human synoviocytes induces inflammation. Exp Biol Med (Maywood). (2019) 244:344-351. doi:10.1177/1535370219830665”.

Reference “35” was erroneously written as “Korbee CJ, Lamers GE, Tengeler AC, Hosseini R, Haks MC, Ottenhoff TH, et al. The DNA damage-regulated autophagy modulator DRAM1 links mycobacterial recognition via TLR-MYD88 to autophagic defense “corrected. Cell Host Microbe. (2014) 15:753–67. doi: 10.1016/j.chom.2014.05.005”. It should be “Kanevets U, Sharma K, Dresser K, Shi Y. A role of IgM antibodies in monosodium urate crystal formation and associated adjuvanticity. J Immunol. (2009) 182:1912-1918. doi:10.4049/jimmunol.0803777”.

Reference “37” was erroneously written as “Macri C, Chin WJ, Panozza SE, Segura E, Patterson NL, Zeller P, et al. Differential use of autophagy by primary dendritic cells specialized in cross-presentation. Autophagy. (2015) 11:906–17. doi: 10.1080/15548627.2015.1045178”. It should be “Saitoh T, Akira S. Regulation of inflammasomes by autophagy. J Allergy Clin Immunol. (2016) 138:28-36. doi:10.1016/j.jaci.2016.05.009”.

Reference “38” was erroneously written as “Jones SA, Ní Cheallaigh C, Hearnden CA, Williams L, Winter J, Lavelle EC, et al. Autophagy regulates IL-23 secretion and innate T cell responses through effects on IL-1 secretion. J Immunol. (2012) 189:41444153. doi: 10.4049/fimmunol.1201946”. It should be “Jones SA, Ní Cheallaigh C, Hearnden CA, Williams L, Winter J, Lavelle EC, et al. Autophagy regulates IL-23 secretion and innate T cell responses through effects on IL-1 secretion. J Immunol. (2012) 189:41444153. doi: 10.4049/jimmunol.1201946”.

Reference “40” was erroneously written as “Fujita N, Jang MH, Uematsu S, Yang BG, Satoh T, Omori H, et al. Loss of the autophagy protein Atgl6Ll enhances endotoxin-induced IL-1beta production. Nature. (2008) 456:264–8. doi: 10.1038/mature07383”. It should be “Fujita N, Jang MH, Uematsu S, Yang BG, Satoh T, Omori H, et al. Loss of the autophagy protein Atgl6Ll enhances endotoxin-induced IL-1beta production. Nature. (2008) 456:264–8. doi: 10.1038/nature07383”.

Reference “41” was erroneously written as “Bhat OM, Meng N, Lohner H, and Li PL. Protective role of autophagy in Nlrp3 inflammasome activation and medial thickening of mouse coronary arteries. Am J Pathol. (2018) 188:2948–59. doi: 10.1016/i.aipath,2018.08.014”. It should be “Bhat OM, Meng N, Lohner H, and Li PL. Protective role of autophagy in Nlrp3 inflammasome activation and medial thickening of mouse coronary arteries. Am J Pathol. (2018) 188:2948–59. doi: 10.1016/j.ajpath.2018.08.014”.

Reference “43” was erroneously written as “Kambas K, Chrysanthopoulou A, Skendros P, Apostolidou E, Kourtzelis I, Drosos GI, et al. Neutrophil extracellular trap formation is associated with IL-lβ and autophagy-related signaling in gout. PloS One. (2011) 6:e29318. doi: 10.1371/ournal.pone.0029318”. It should be “Kambas K, Chrysanthopoulou A, Skendros P, Apostolidou E, Kourtzelis I, Drosos GI, et al. Neutrophil extracellular trap formation is associated with IL-lβ and autophagy-related signaling in gout. PloS One. (2011) 6:e29318. doi: 10.1371/journal.pone.0029318”.

Reference “44” was erroneously written as “Shen M, McDonald C, and Yao Q. Autophagy dysfunction in autoinflammatory diseases. Autoimmun. (2018) 88:1 1–20. doi: 10.1016/i.jaut.2017.10.012”. It should be “Shen M, McDonald C, and Yao Q. Autophagy dysfunction in autoinflammatory diseases. Autoimmun. (2018) 88:1 1–20. doi: 10.1016/j.jaut.2017.10.012”.

Reference “45” was erroneously written as “Yu QH, Chu Y, Wu WM, Song JX, Zhu XB, Wang Q, et al. Blockage of AKAP12 accelerate angiotensin l (Ang)-induced cardiae injury in mice by regulating the transforming growth factor B: (TGF-β) pathway. Biochem Bioph Res Co. (2018) 499:128–35”. It should be “Hwang HS, Yang CM, Park SJ, Kim HA. Monosodium Urate Crystal-Induced Chondrocyte Death via Autophagic Process. Int J Mol Sci. (2015) 16:29265-29277. Published 2015 Dec 8. doi:10.3390/ijms161226164”.

Reference “48” was erroneously written as “He YL, Zhong XW, Xie WG, and Zhou JG. Resveratrol ameliorates inflammation via upregulation of sirtuin 1 to promote autophagy in gout patients. Inflammopharmacology. (2019) 27:47–56. doi: 10.1007/s10787-018-00555-4”. It should be “Shi CS, Shenderov K, Huang NN, et al. Activation of autophagy by inflammatory signals limits IL-1β production by targeting ubiquitinated inflammasomes for destruction. Nat Immunol. (2012) 13:255-263. Published 2012 Jan 29. doi:10.1038/ni.2215”.

Reference “49” was erroneously written as “Chen S, Qian H, Zheng Q, Chen R, Liu Y, Shi G, et al. Role of T cells in the pathogenesis and treatment of gout. Int Immunopharmacol. (2020) 88:106877–89. doi: 10.1016/j.intimp.2020.106877”. It should be “Choe JY, Jung HY, Park KY, Kim SK. Enhanced p62 expression through impaired proteasomal degradation is involved in caspase-1 activation in monosodium urate crystal-induced interleukin-1b expression. Rheumatology (Oxford). (2014) 53:1043-1053. doi:10.1093/rheumatology/ket474”.

Reference “50” was erroneously written as “Chen W, Zhang X, Mei L, Wu X, Chen X, Yang Z, et al. Single-cell analysis in blood reveals distinct immune cell profiles in gouty arthritis. J Immunol. (2023) 210:745–52. doi: 10.4049/jimmunol.2200422”. It should be “Yang QB, He YL, Zhong XW, Xie WG, Zhou JG. Resveratrol ameliorates gouty inflammation via upregulation of sirtuin 1 to promote autophagy in gout patients. Inflammopharmacology. (2019) 27:47-56. doi:10.1007/s10787-018-00555-4”.

Reference “51” was erroneously written as “Choi SJ, Ahn SM, Oh JS, Kim YG, Lee CK, Yoo B, et al. Neutrophil extracellular trap clearance by synovial macrophages in gout. Arthritis Res Ther. (2021) 23:88–98. doi: 10.1186/s13075-021-02472-4”. It should be “Tausche AK, Richter K, Grässler A, Hänsel S, Roch B, Schröder HE. Severe gouty arthritis refractory to anti-inflammatory drugs: treatment with anti-tumour necrosis factor alpha as a new therapeutic option. Ann Rheum Dis. (2004) 63:1351-1352. doi:10.1136/ard.2003.015743”.

Reference “52” was erroneously written as “Raucci F, Saviano A, Tull S, Maione F, and Iqbal AJ. Galectin-9 regulates monosodium urate crystal-induced gouty inflammation through the modulation of Treg/Th17 ratio. Front Immunol. (2021) 28:762016–27. doi: 10.3389/fimmu.2021.762016”. It should be “Zhang Y, Pan R, Xu Y, Zhao Y. Treatment of refractory gout with TNF-α antagonist etanercept combined with febuxostat. Ann Palliat Med. (2020) 9:4332-4338. doi:10.21037/apm-20-2072”.

Reference“53” was erroneously written as “Calvo-Aranda E and Sanchez-Aranda FM Efficacy of subcutaneous tocilizumab in a patient with severe gout refractory to anakinra. Rheumatol (Oxford). (2021) 60:e375–7. doi: 10.1093/rheumatology/keab383”. It should be “Sumiyoshi R, Koga T, Tsuji S, et al. Chlamydia-induced reactive arthritis diagnosed during gout flares: A case report and cumulative effect of inflammatory cytokines on chronic arthritis. Medicine (Baltimore). (2019);98:e17233. doi:10.1097/MD.0000000000017233”.

Reference “54” was erroneously written as “Andrés M, Vela P, and Pascual E. Interleukin-6 pathway blockade as an option for managing refractory cases of crystal arthritis: two cases report. Joint Bone Spine. (2022) 85:377–8. doi: 10.1016/j.JBSPIN.2022.04008”. It should be “Fiehn C, Zeier M. Successful treatment of chronic tophaceous gout with infliximab (Remicade). Rheumatol Int. (2006) 26:274-276. doi:10.1007/s00296-005-0617-7”.

Reference “55” was erroneously written as “Ea HK, Frazier A, Blanchard A, Lioté F, Marotte H, Bardin T, et al. Tocilizumab in symptomatic calcium pyrophosphate deposition disease: a pilot study. Ann Rheum Dis. (2020) 79:1126–8. doi: 10.1136/annrheumdis-2020-217188”. It should be “Pinto JL, Mora GE, Fernández-Avila DG, Gutiérrez JM, Díaz MC; Grupo Javeriano de Investigación en Enfermedades Reumáticas. Tocilizumab in a patient with tophaceous gout resistant to treatment. Reumatol Clin. (2013) 9:178-180. doi:10.1016/j.reuma.2012.06.009”.

Reference “56” was erroneously written as “Bian M, Zhu C, Nie A, and Zhou Z Guizhi Shaoyao Zhimu Decoction ameliorates gouty arthritis in rats via altering gut microbiota and improving metabolic profile. Phytomedicine. (2024) 131:155800. doi: 10.1016/j.phymed.2024.15580”. It should be “Mokuda S, Kanno M, Takasugi K, Okumura C, Ito Y, Masumoto J. Tocilizumab improved clinical symptoms of a patient with systemic tophaceous gout who had symmetric polyarthritis and fever: An alternative treatment by blockade of interleukin-6 signaling. SAGE Open Med Case Rep. 2014 Jan 8;2:2050313X13519774. doi: 10.1177/2050313X13519774”.

Reference “57” was erroneously written as “Gut microbiota remodeling: A promising therapeutic strategy to confront hyperuricemia and gout. Front Cell Infect Microbiol. (2022) 12:935723. doi: 10.3389/fcimb.2022.935723”. It should be “Calvo-Aranda E, Sanchez-Aranda FM. Efficacy of subcutaneous tocilizumab in a patient with severe gout refractory to anakinra. Rheumatology (Oxford). (2021) 60:e375-e377. doi:10.1093/rheumatology/keab383”.

Reference “58” was erroneously written as “Shirvani-Rad S, Khatibzade-Nasari N, Ejtahed HS, and Larijani B Exploring the role of gut microbiota dysbiosis in gout pathogenesis: a systematic review. Front Med (Lausanne). (2023) 10:1163778. doi: 10.3389/fmed.2023.1163778”. It should be “Pers YM, Schaub R, Constant E, et al. Efficacy and safety of tocilizumab in elderly patients with rheumatoid arthritis. Joint Bone Spine. (2015) 82:25-30. doi:10.1016/j.jbspin.2014.07.010”.

Reference “59” was erroneously written as “Guo Z, Zhang J, Wang Z, Ang KY, Huang S, Hou Q, et al. Intestinal microbiota distinguish gout patients from healthy humans. Sci Rep. (2016) 6:20602. doi: 10.1038/srep20602”. It should be “Latourte A, Ea HK, Frazier A, et al. Tocilizumab in symptomatic calcium pyrophosphate deposition disease: a pilot study. Ann Rheum Dis. (2020) 79:1126-1128. doi:10.1136/annrheumdis-2020-217188”.

Reference “66” was erroneously written as “Xiao X, Shi X, Fan Y, Zhang X, Wu M, Lan P, et al. GITR subverts Foxp3(+) Tregs to boost Th9 immunity through regulation of histone acetylation. Nat Commun. (2015) 6:8266. doi: 10.1038/ncomms9266”. It should be “Liu P, Hu P, Jin M, et al. Compound probiotics alleviate hyperuricemia-induced renal injury via restoring gut microbiota and metabolism. BMC Microbiol. (2025) 25:280. Published 2025 May 8. doi:10.1186/s12866-025-04012-5”.

Reference “69” was erroneously written as “Yang Q, Zhang Q, Qing Y, Zhou L, Mi Q, and Zhou J miR-155 is dispensable in monosodium urate-induced gouty inflammation in mice. Arthritis Res Ther. (2018) 20:144. doi: 10.1186/s13075-018-1550-y”. It should be “Xiao Y, Li B, Zhou Z, Hancock WW, Zhang H, Greene MI. Histone acetyltransferase mediated regulation of FOXP3 acetylation and Treg function. Curr Opin Immunol. (2010) 22:583-591. doi:10.1016/j.coi.2010.08.013”.

Reference “70” was erroneously written as “Jin HM, Kim TJ, Choi JH, Kim MJ, Cho YN, Nam KI, et al. MicroRNA-155 as a proinflammatory regulator via SHIP-1 down-regulation in acute gouty arthritis. Arthritis Res Ther. (2014) 16:R88. doi: 10.1186/ar4531”. It should be “Deng G, Song X, Fujimoto S, Piccirillo CA, Nagai Y, Greene MI. Foxp3 Post-translational Modifications and Treg Suppressive Activity. Front Immunol. (2019) 10:2486. doi:10.3389/fimmu.2019.02486”.

Reference “71” was erroneously written as “Song S, Shi K, Fan M, Wen X, Li J, Guo Y, et al. Clostridium butyricum and its metabolites regulate macrophage polarization through miR-146a to antagonize gouty arthritis. J Adv Res. (2025). doi: 10.1016/j.jare.2025.05.036”. It should be “Badii M, Gaal OI, Cleophas MC, et al. Urate-induced epigenetic modifications in myeloid cells. Arthritis Res Ther. (2021) 23:202. doi:10.1186/s13075-021-02580-1”.

Reference “72” was erroneously written as “Zhang QB, Qing YF, Yin CC, Zhou L, Liu XS, Mi QS, et al. Mice with miR-146a deficiency develop severe gouty arthritis via dysregulation of TRAF 6, IRAK 1 and NALP3 inflammasome. Arthritis Res Ther. (2018) 20:45. doi: 10.1186/s13075-018-1546-7”. It should be “Straton AR, Kischkel B, Crişan TO, Joosten LAB. Epigenomic Reprogramming in Gout. Gout, Urate, and Crystal Deposition Disease. (2024) 2:325-338. https://doi.org/10.3390/gucdd2040023”.

Reference “73” was erroneously written as “Yarwood H, Harrison AA, Stocker CJ, Jamar F, Gundel RH, Peters AM, et al. Endothelial activation in monosodium urate monohydrate crystal-induced inflammation: *in vitro* and *in vivo* studies on the roles of tumor necrosis factor alpha and interleukin-1. Arthritis Rheum. (1997) 40:955–65. doi: 10.1002/art.1780400525”. It should be “Yang Q, Zhang Q, Qing Y, Zhou L, Mi Q, Zhou J. Correction to: miR-155 is dispensable in monosodium urate-induced gouty inflammation in mice. Arthritis Res Ther. (2018) 20:177. doi:10.1186/s13075-018-1696-7”.

Reference “74” was erroneously written as “Yoshimura T, Maeda T, Takahashi T, Ohkawara S, and Yoshinaga M. Analysis of the cytokine network among tumor necrosis factor alpha, interleukin-1beta, interleukin-8, and interleukin-l receptor antagonist in monosodium urate crystal-induced rabbit arthritis. Lab Invest. (1998) 78:559–69. doi: 10.1121/1.422796”. It should be “Jin HM, Kim TJ, Choi JH, et al. MicroRNA-155 as a proinflammatory regulator via SHIP-1 down-regulation in acute gouty arthritis. Arthritis Res Ther. (2014) 16:R88. doi:10.1186/ar4531”.

Reference “75” was erroneously written as “Schmid D, Pypaert M, and Minz C Antigen-loading compartments for major histocompatibility complex class molecules continuously receive input from autophagosomes. Immunity. (2007) 26:79–92. doi: 10.1016/f.immuni.2006.10.018”. It should be “Song S, Shi K, Fan M, et al. Clostridium butyricum and its metabolites regulate macrophage polarization through miR-146a to antagonize gouty arthritis. J Adv Res. doi:10.1016/j.jare.2025.05.036”.

Reference “76” was erroneously written as “Kasahara K, Kerby RL, Zhang Q, Pradhan M, Mehrabian M, Lusis AJ, et al. Gut bacterial metabolism contributes to host global purine homeostasis. Cell Host Microbe. (2023) 31:1038–1053.e10. doi: 10.1016/j.chom.2023.05.011”. It should be “Zhang QB, Qing YF, Yin CC, et al. Mice with miR-146a deficiency develop severe gouty arthritis via dysregulation of TRAF 6, IRAK 1 and NALP3 inflammasome. Arthritis Res Ther. (2018) 20:45. doi:10.1186/s13075-018-1546-7”.

Also, there were errors in-text citations. The names in the main text were inconsistent with the references listed below:

In section **2 Immunological pathogenesis of gouty arthritis**, *2.1.3 Monocyte-to-macrophage differentiation and inflammation resolution* , 4. This sentence previously stated: “Research by Darshna et al. demonstrated that partially differentiated macrophages are characterized by high TNF-α secretion and robust ICAM-1 induction, both of which can be attenuated through TNF-α blockade. Fully differentiated macrophages, on the other hand, predominantly function in MSU crystal clearance with minimal cytokine output (23)”. The corrected sentence is: “Research by So et al. demonstrated that partially differentiated macrophages are characterized by high TNF-α secretion and robust ICAM-1 induction, both of which can be attenuated through TNF-α blockade. Fully differentiated macrophages, on the other hand, predominantly function in MSU crystal clearance with minimal cytokine output (23)”

In section **2 Immunological pathogenesis of gouty arthritis**, *2.3 Cytokine-mediated regulation of gouty inflammation* , 2. This sentence previously stated: “Lioté et al. reported that recombinant human TGF-β1 significantly reduced MSU-induced leukocyte infiltration and monocyte accumulation. TGF-β1 antagonists reversed these effects, validating its anti-inflammatory function. Additionally, MSU crystals upregulate monocyte/macrophage-derived TGF-β, suppressing neutrophil recruitment via E-selectin inhibition (25)”. The corrected sentence is: “Shi et al. reported that recombinant human TGF-β1 significantly reduced MSU-induced leukocyte infiltration and monocyte accumulation. TGF-β1 antagonists reversed these effects, validating its anti-inflammatory function. Additionally, MSU crystals upregulate monocyte/macrophage-derived TGF-β, suppressing neutrophil recruitment via E-selectin inhibition (25)”

In section **2 Immunological pathogenesis of gouty arthritis**, *2.3 Cytokine-mediated regulation of gouty inflammation* , 5. This sentence previously stated: “Chapman et al. demonstrated that TNF-α blockade reduces E-selectin expression and neutrophil recruitment in MSU-induced arthritis”. The corrected sentence is: “Busso et al. demonstrated that TNF-α blockade reduces E-selectin expression and neutrophil recruitment in MSU-induced arthritis”

In section **2 Immunological pathogenesis of gouty arthritis**, *2.3 Cytokine-mediated regulation of gouty inflammation* , 8. This sentence previously stated: “Yokose et al. revealed TNF-α-driven neutrophil activation, caspase-1 secretion, and subsequent IL-1β cleavage and activation, further amplifying inflammatory responses”. The corrected sentence is: “Stefanie et al. revealed TNF-α-driven neutrophil activation, caspase-1 secretion, and subsequent IL-1β cleavage and activation, further amplifying inflammatory responses”.

Lastly, as some of the references cited in the sections below were less relevant, they have been replaced with more suitable sources.

A correction has been made to [2 Immunological pathogenesis of gouty arthritis], [2.5 Adaptive immunity in GA pathogenesis], [2]. This sentence previously stated:

“[Recent investigations have revealed that monosodium urate (MSU) crystals can directly engage CD8+ T lymphocytes, inducing enhanced phagocytic function that contributes to the pro-inflammatory microenvironment characteristic of gouty arthritis (GA) (33)]”

The corrected sentence appears below:

“[MSU crystals can directly participate in immune cells and contribute to gouty arthritis (GA) (33)]”

A correction has been made to [4 Therapeutic immunomodulation in gouty arthritis], [4.2 IL-6 pathway inhibition in refractory gout management], [8]. This sentence previously stated:

“[Similarly, Ouilis et al. (58) reported two geriatric cases where TCZ effectively managed crystal-induced arthritis after anakinra discontinuation due to adverse effects, demonstrating persistent clinical improvement and biomarker normalization during 9–11 months of observation]”

The corrected sentence appears below:

“[Pers et al. (58) evaluated tocilizumab (TCZ) in elderly rheumatoid arthritis patients, finding that while it was well tolerated, its efficacy was lower compared to younger patients. Despite this, TCZ led to sustained clinical improvement over 6 months with good tolerability.]”

The original version of this article has been updated.

